# Apoptosis-Inducing and Proliferation-Inhibiting Effects of Doramectin on Mz-ChA-1 Human Cholangiocarcinoma Cells

**DOI:** 10.3390/ijms252413440

**Published:** 2024-12-15

**Authors:** Yunfang Zhang, Wei Wu, Yan Shi, Yuehong Huang, Ting Dai, Lina Ke, Lizhu Chen, Mingliang Chen, Qin Wang

**Affiliations:** 1 State Key Laboratory of Cellular Stress Biology, School of Life Sciences, Xiamen University, Xiamen 361102, China; 15260237644@163.com (Y.Z.); victorwuwei@outlook.com (W.W.); yshi@xmu.edu.cn (Y.S.); 21620221153418@stu.xmu.edu.cn (Y.H.); 21620231153389@stu.xmu.edu.cn (T.D.); kerlinda@xmu.edu.cn (L.K.); clz0823@xmu.edu.cn (L.C.); 2 Key Laboratory of Marine Genetic Resources, Third Institute of Oceanography, Ministry of Natural Resources, Xiamen 361005, China; 3 Co-Innovation Center of Jiangsu Marine Bio-Industry Technology, Jiangsu Ocean University, Lianyungang 222000, China

**Keywords:** doramectin, Mz-ChA-1, apoptosis, proliferation-inhibiting

## Abstract

Cholangiocarcinoma is a malignant tumor that emerges in the intrahepatic or extrahepatic bile ducts. Doramectin (DOR), a third-generation derivative of avermectins (AVMs), is renowned for its low toxicity and high efficiency. However, no research has hitherto focused on the anti-cholangiocarcinoma effects of these drugs. In this study, we undertook a preliminary exploration of the mechanism through which DOR inhibits the viability of human cholangiocarcinoma cells (Mz-ChA-1) via transcriptome analysis and molecular validation at the cellular level. The results indicated that DOR could suppress the growth and proliferation of Mz-ChA-1 cells in a dose-dependent manner. Moreover, it significantly diminished their migration and invasion abilities. Cell cycle analysis disclosed arrest in the G1 phase, accompanied by an increase in p21 expression and a decrease in the levels of the cyclin E1 and CDK2 proteins. Additionally, DOR induced apoptosis via the ROS-triggered mitochondrial pathway. This was attested by an elevation in the BAX/BCL-2 ratio, the activation of caspase 3/7 and the cleavage of PARP1. These mechanistic insights underscore DOR’s potential as a therapeutic agent against cholangiocarcinoma

## 1. Introduction

Cholangiocarcinomas (CCA) are a heterogeneous group of malignant tumors that can arise at every point in the biliary tree [1,2]. They are the second most common primary liver malignancy, following hepatocellular carcinoma, accounting for 10–20% of all hepatic cancers [3,4]. A considerable number of individuals diagnosed with cholangiocarcinoma are without symptoms, and their tumors are fortuitously identified in routine imaging examinations. At the point of diagnosis, the majority of individuals have unresectable stage III or stage IV disease [5]. At present, surgery remains the primary treatment of CCA, along with radiotherapy and chemotherapy [6,7]. However, due to advanced local tumor infiltration or peritoneal or distant metastases, lack of biliary reconstruction options and insufficient future liver remnants, less than one third of patients are classified as having resectable tumors at diagnosis [8]. Several targeted therapies with potential therapeutic benefits have been developed and have shown encouraging response rates and survival rates as second-line treatments. They include those targeting the fibroblast growth factor receptor (FGFR), isocitrate dehydrogenase, the RAS-RAF-MEK signaling pathway, human epidermal growth factor receptor 2 and neurotrophic receptor tyrosine kinase [9]. Among them, FGFR inhibitors have shown high remission rates in the treatment of patients with FGFR fusions/rearrangements and FGFR mutations [10,11,12]. However, the side effects, especially hyperphosphatemia, remain a concern [13]. These observations emphasize the need to develop effective therapies against CCA.

Avermectins are 16-membered cyclolides produced by the fermentation of *Streptomyces avermitilis*. According to its structural characteristics, AVM is divided into eight components: A1a, A1b, A2a, A2b, B1a, B1b, B2a and B2b. Among them, B1 (B1a and B1b) are the main active ingredients. Avermectin drugs include avermectin, ivermectin, doramectin, eprovermectin, moxectin and seramectin [14]. They are considered as potential neurotoxins that target gamma aminobutyric acid receptors and glutamate-gated chloride channels [15,16,17], which in mammals are mainly located in the brain protected by the blood–brain barrier, and are therefore commonly used as insecticides for the treatment of pests and parasites [18,19]. DOR is a genetically modified AVM produced by actinomycetes (fungi) with potent repellent and insecticidal activity [20]. Recent studies have shown that DOR can induce apoptosis and necroptosis in glioma cells, which is expected to be a potential anticancer drug [21,22]. Although there are few reports of clinical trials on DOR in humans, there are reports that ivermectin, which is also a member of the avermectin family, is well tolerated at doses of up to 300 μg/kg [23]. Therefore, analyzing the mechanism of action of DOR is still of theoretical value in the search for safe and effective anticancer drugs.

In this paper, we selected the human cholangiocarcinoma cell line Mz-ChA-1 as our research model. We revealed the inhibitory effect of DOR on Mz-ChA-1 cells and explored the related mechanisms. This study provides new insights and basic theoretical support for the development of anti-cholangiocarcinoma drugs.

## 2. Results

### 2.1. DOR Inhibits Mz-ChA-1 Cell Proliferation and Cell Migration Capacity

**Figure 1:**
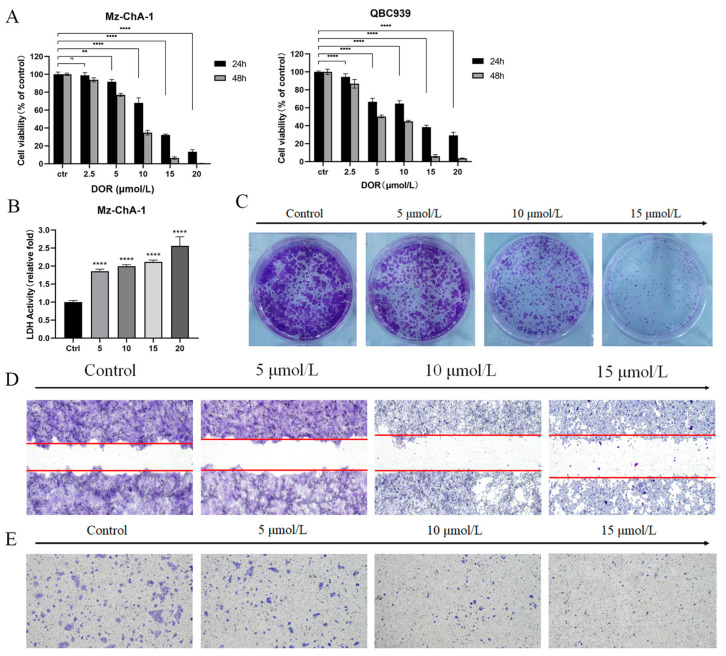
DOR inhibits Mz-ChA-1 cell proliferation and cell migration capacity. (**A**) Mz-ChA-1 and QBC939 cells were treated with different doses of DOR for 24 h and 48 h, after which cell viability was determined via the CCK-8 assay. (**B**) Mz-ChA-1 cells were incubated with different doses of DOR for 48 h. Results show the cellular LDH release detected by the LDH assay kit. (**C**) Analysis of Mz-ChA-1 cell colonies by crystal violet staining after a 48 h exposure to varying concentrations of DOR. (**D**) Mz-ChA-1 cells were treated with different doses of DOR for 24 h. Giemsa staining was performed to observe the filling of scratches by the cells. The red line distinguishes the border between the two ends of the scratch. (**E**) Mz-ChA-1 cells were treated with different doses of DOR for 24 h. The Transwell assay shows the effect of DOR on cell invasion ability. Significance levels were categorized as: ns: not significant, ** *p* < 0.01 and **** *p* < 0.0001.

To evaluate the inhibitory effect of DOR on cholangiocarcinoma cells, Mz-ChA-1 and QBC939 cells were treated with different concentrations of DOR for 24 h and 48 h, respectively. The results showed that the cell viabilities of Mz-ChA-1 and QBC939 were reduced with increasing concentration and treatment time, and the inhibition rate reached 60% after 10 μmol/L DOR treatment for 24 h (Figure 1A). The half-inhibitory concentrations (IC_50_) of the drug at these two time points (24 h and 48 h) were calculated, by plotting the fitting curves, to be 12.16 μmol/L and 7.613 μmol/L, while the IC_50_s of QBC939 were 11.52 μmol/L and 6.035 μmol/L. Therefore, the concentration gradients of 0, 5, 10 and 15 μmol/L were used to investigate the inhibition efficiency of DOR on Mz-ChA-1 cells. The colony-forming ability of cells decreased with increasing DOR dose, while the concentration of lactate dehydrogenase (LDH) showed an increasing trend, suggesting that DOR not only affects cell proliferation, but also promotes Mz-ChA-1 death at the same time (Figure 1B,C). In addition, the results from the cell scratch assay and Transwell chambers showed that the migration and invasion ability of Mz-ChA-1 cells were inhibited by DOR treatment (Figure 1D,E). These results suggest that DOR may have multiple inhibitory effects on Mz-ChA-1 cells, rather than a single inhibition of tumor cell proliferation or migration and invasion ability.

We examined two normal cell lines of human origin (Hacat and AC16). The results showed that these normal cell lines exhibited a similar reduction in cell viability as the cholangiocarcinoma cell lines at high doses of DOR, but no significant inhibition of cell viability was found in these cells at low concentrations of DOR (Figure S1).

### 2.2. DOR Inhibits G1/S Phase Transition in Mz-ChA-1 Cells

**Figure 2:**
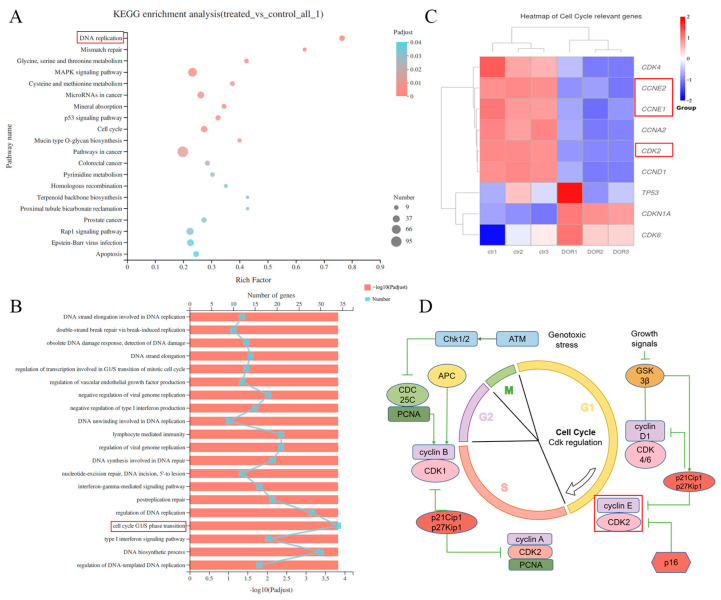
DOR inhibits G1/S phase transition in Mz-ChA-1 cells. (**A**) Bubble plot showing significantly changed signaling pathways enriched by KEGG enrichment analysis after DOR (15 μmol/L) treatment (*n* = 3). The red box highlights one of the pathways involved in DNA replication after KEGG enrichment analysis. (**B**) Bar line showing significantly changed cellular processes enriched by GO (gene ontology) enrichment analysis after DOR (15 μmol/L) treatment (*n* = 3). The red box highlights one of the GO enrichment results related to the cellular process of the G1/S phase transition. (**C**) Heatmap showing expression of cell cycle-related genes after DOR (15 μmol/L) treatment (*n* = 3). Red boxes highlight the expression of the *CCNE1*, *CCNE2* and *CDK2* genes (**D**) The cell cycle diagram shows the phases in which the major key enzymes perform their functions. The red box highlights the cell cycle period in which the cyclin E and CDK2 proteins are functioning.

In order to identify the intrinsic mechanism by which DOR inhibits the viability of Mz-ChA-1 cells, transcriptome analysis was performed on DOR-treated cells. Kyoto Encyclopedia of Genes and Genomes (KEGG) enrichment analysis showed that the most significant changes in DOR-treated Mz-ChA-1 cells were in the pathway associated with DNA replication (Figure 2A). In this pathway, the genes that underwent significant changes were mainly involved in the G1/S phase transition, which included cyclin E1 *(CCNE1)*, cyclin E2 *(CCNE2)* and *CDK2* (Figure 2B,C). The major kinases involved in cell cycle transitions and their associated proteins are shown in Figure 2D.

### 2.3. DOR Inhibits Cyclin E1 and CDK2 Gene Expression in Mz-ChA-1 Cells

**Figure 3:**
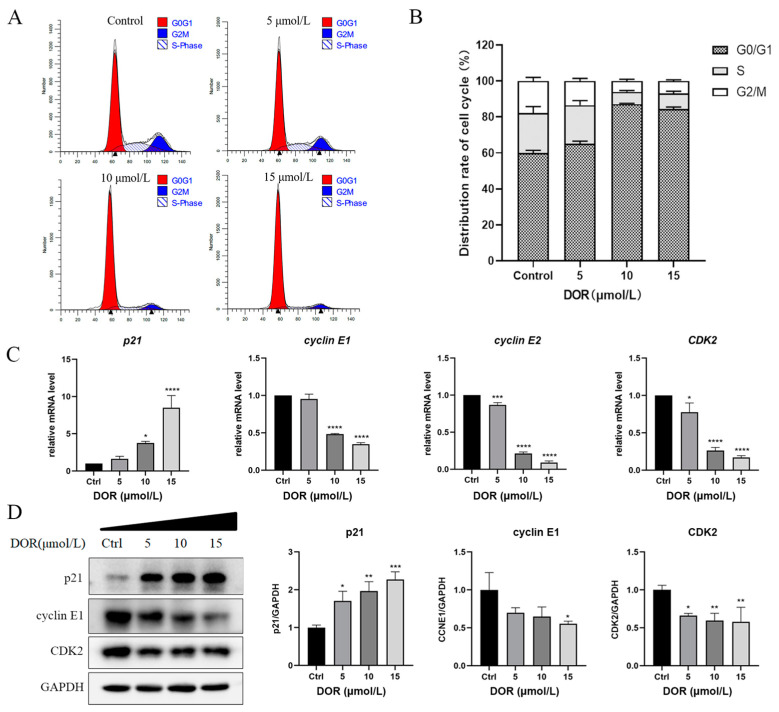
DOR inhibits cyclin E1 and CDK2 gene expression in Mz-ChA-1 cells. (**A**,**B**) Mz-ChA-1 cells exposed to varying doses of DOR for 24 h were labeled with propidine iodide (PI) and detected by flow cytometry (*n* = 3). A representative cell cycle diagram is shown in (**A**). (**C**) Mz-ChA-1 cells were treated with varying doses of DOR for 24 h. The bar graph shows the transcription results of cell cycle-related genes (*p21*, *CDK2*, *cyclin E1* and *cyclin E2*) detected by RT-qPCR. (**D**) Mz-ChA-1 cells were treated with varying doses of DOR for 24 h (*n* = 3). Western blot analysis delineated the expression profiles of cell cycle-regulatory proteins, including p21, cyclin E1 and CDK2. Significance levels were categorized as: * *p* < 0.05, ** *p* < 0.01, *** *p* < 0.001 and **** *p* < 0.0001.

After analyzing potential pathways of action using transcriptome sequencing, the results were verified by flow cytometry, RT-qPCR and Western blot experiments. Flow cytometry results showed that the number of S and G2 cells did decrease significantly after 24 h of DOR treatment (Figure 3A). With the increase of DOR concentration, the proportion of G0/G1 period cells increased from 60% at 5 μmol/L to 90% at 10 μmol/L, indicating that the cells were blocked in the G1 phase (Figure 3B). The expression of cyclin E1 and CDK2 was detected to decrease with increasing DOR concentration at both the mRNA level and protein level, whereas the expression of p21 increased significantly (Figure 3C,D).

### 2.4. DOR Induces Apoptosis in Mz-ChA-1 Cells

**Figure 4:**
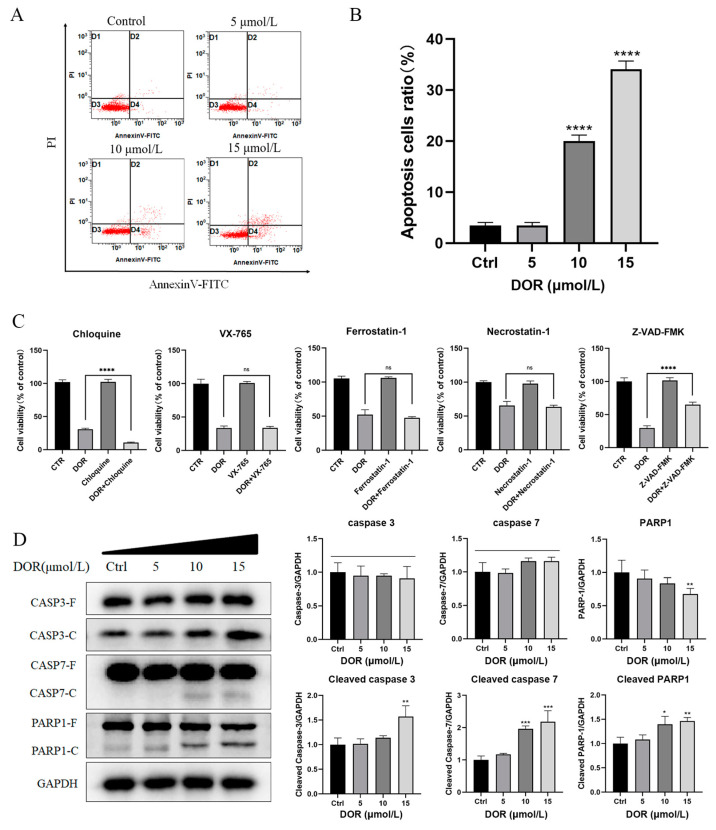
DOR induces apoptosis in Mz-ChA-1 cells. (**A**,**B**) Mz-ChA-1 cells treated with DOR at varying concentrations for 48 h were subjected to Annexin V-FITC/PI staining, followed by flow cytometry analysis (*n* = 3). (**A**) illustrates a typical density plot from the Annexin V-FITC/PI staining. (**C**) Effect of DOR (10 μmol/L) on the proliferation of Mz-ChA-1 cells for 48 h after pretreatment with inhibitors. Inhibitors included: chloroquine (cellular autophagy inhibitor, 20 μM), VX-765 (specific inhibitor of caspase-1, 50 μM), ferrostatin-1 (iron death inhibitor, 0.5 μM), necrostatin-1 (necrotic pathway inhibitor, 30 μM) and Z-VAD-FMK (apoptosis pathway inhibitor, 20 μM). *n* = 4. (**D**) Mz-ChA-1 cells were treated with different doses of DOR for 24 h. Western blot analysis revealed the cleavage of apoptosis-related proteins. CASP3-F/C indicates caspase 3 full-length/cleavage protein; CASP7-F/C indicates caspase 7 full-length protein/cleavage protein; PARP1-F/C indicates PARP1 full-length protein/cleaved protein. Significance levels were categorized as: ns: not significant, *p* > 0.05, * *p* < 0.05, ** *p* < 0.01, *** *p* < 0.001 and **** *p* < 0.0001.

In addition to the occurrence of proliferation inhibition, apoptosis was also detected in DOR-treated Mz-ChA-1 cells (Figure 4A). The percentage of apoptotic cells reached 20% after 48 h of DOR treatment at 10 μmol/L (Figure 4B). In inhibitor treatment experiments, pretreatment with Z-VAD-FMK, a broad-spectrum caspase inhibitor, significantly restored the reduced cell viability of Mz-ChA-1 cells after DOR treatment (Figure 4C). In addition, cleavage of apoptosis-associated proteins caspase 3 and caspase 7, two caspase family members that are key proteins in the apoptotic pathway, were detected in DOR-treated cells (Figure 4D). The levels of cleaved caspase 3 and caspase 7 were up-regulated with the increasing DOR concentrations. PARP1, another protein related to apoptosis, was also observed to be cleaved after DOR treatment (Figure 4D). The cleavage of PARP1 by caspase 3 and caspase 7, which resulted in the inhibition of DNA repair [24], is a significant indicator of apoptosis [25].

### 2.5. DOR Inhibits the Expression of the Antiapoptotic Protein BCL-2

**Figure 5:**
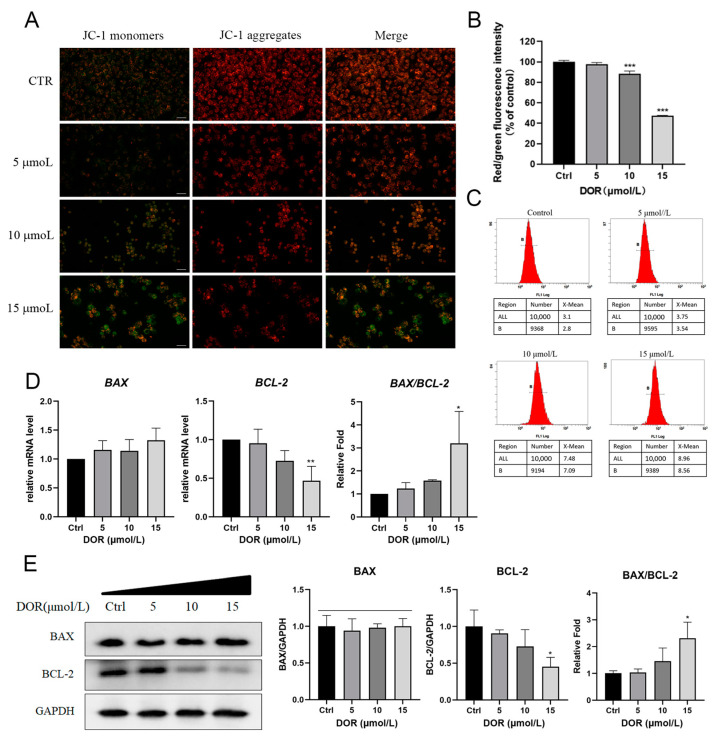
DOR inhibits the expression of the antiapoptotic protein BCL-2. (**A**) Fluorescence plot of mitochondrial transmembrane potential changes in Mz-ChA-1 cells after DOR action. Scale bar, 25μm. (**B**) Red-to-green fluorescence ratio (*n* = 3). (**C**) Changes in ROS in Mz-ChA-1 cells treated with different concentrations of DOR. (**D**) Mz-ChA-1 cells were treated with different doses of DOR for 24 h. The bar graph shows the transcription results of *BAX*, *BCL-2* and their relative ratio detected by RT-qPCR. (**E**) Mz-ChA-1 cells were treated with different doses of DOR for 24 h. The results of Western blot show the expression of BAX and BCL-2. Significance levels were categorized as: * *p* < 0.05, ** *p* < 0.01, *** *p* < 0.001.

Apoptotic pathways are usually activated by intrinsic or extrinsic pathways. Considering the large number of gene expressions associated with DNA damage repair detected in the transcriptome sequencing results (Figure 2B), it is more likely that the intrinsic pathway plays a key role in DOR-induced apoptosis [26]. In the intrinsic apoptotic pathway, partial permeability of the mitochondrial membrane disrupts the balance of mitochondrial ions and volume, resulting in the loss of the mitochondrial transmembrane potential (DeltaPsi(m)) [27]. A JC-1 staining assay allowed the testing of whether DNA damage stress induced by DOR treatment triggered changes in mitochondrial membrane voltage [28]. Fluorescence microscopy showed that the red/green fluorescence intensity decreased with increasing DOR concentrations, suggesting that mitochondrial membrane potential decreased in a dose-dependent manner (Figure 5A,B). In addition, the 2′,7′-dichlorodihydrofluorescein diacetate (DCFH-DA) probe assay showed that intracellular reactive oxygen species (ROS) levels were increased (3.17 ± 0.12, 3.73 ± 0.02, 7.58 ± 0.17 and 8.94 ± 0.04) after DOR treatment (0, 5, 10 and 15 μmol/L) (Figure 5C). The above results suggest that DOR-induced apoptosis occurs mainly through the intrinsic pathway.

Imbalances in BCL-2 family protein expression often lead to changes in mitochondrial membrane potential, which is crucial for the intrinsic apoptotic pathway [29,30]. Our results indicated that BCL-2 protein level was decreased significantly with increasing DOR concentration (Figure 5D). As an antiapoptotic protein, the decreased expression of BCL-2 is likely to be one of the reasons for the pro-apoptotic effect of DOR.

## 3. Discussion

In recent years, numerous studies have demonstrated that macrocyclic lactones, including various compounds, can inhibit the viability of cancer cells [31,32,33]. However, the potential of DOR to inhibit cholangiocarcinoma cells has not been fully explored. In this study, we confirmed that DOR effectively inhibits the viability of Mz-ChA-1 cells, a CCA cell line. This inhibitory effect is achieved through a dual mechanism: by suppressing cell proliferation and promoting apoptosis, suggesting that DOR may have potential as a therapeutic agent for CCA.

Most cancer deaths are caused by metastasis, the process by which tumor cells spread from their primary site to distant organs [34,35,36]. During this dissemination, tumor cells carrying oncogenic driver mutations invade deeper tissue layers, often breaching the basement membrane, and gain the ability to survive in the absence of specific growth factors from their native environment [37]. Cell migration and cancer cell metastasis can be effectively simulated using in vitro models such as the cell scratch assay and Transwell assay [38,39]. In this study, the results demonstrated that DOR significantly inhibited the migration and invasion of Mz-ChA-1 cells, suggesting that DOR may have potential anti-metastatic properties in cholangiocarcinoma. However, the precise mechanisms by which DOR exerts these effects on the invasion of cholangiocarcinoma cells remain to be fully elucidated. Future research will be crucial to uncover the molecular pathways involved in this inhibition and to assess the therapeutic potential of DOR in preventing metastasis in CCA.

The transcriptome encompasses all RNA transcripts within a cell, including their quantities, at a given developmental stage or physiological condition. Analyzing the transcriptome is pivotal for revealing genomic functions, mapping cellular and tissue molecules, and probing developmental and pathological processes [40,41]. To further investigate the changes in the transcriptional levels of Mz-ChA-1 cells after DOR treatment, we performed transcriptome sequencing analysis and detected a total of 3349 differentially expressed genes. KEGG enrichment analysis enriched a total of 329 pathways, of which the top 20 pathways included the DNA replication pathway, cell cycle pathway, p53 signaling pathway and apoptosis pathway. GO enrichment analysis enriched a total of 2991 biological processes, among which cell cycle G1/S phase transition was enriched to 34 related genes that changed in expression level after DOR treatment. Our results indicated that the most significant impact of DOR treatment on Mz-ChA-1 cells was on the cell proliferation signaling pathway, followed by the apoptosis pathway.

The development of cancer is related to the disturbance of cell cycle regulation, and there is a close relationship between anti-tumor drugs and cell cycle [42,43]. In mammalian cells, cyclin E1 and cyclin E2 activate CDK2, thereby propelling cell cycle advancement through the phosphorylation of multiple substrates [44,45]. Recent studies have shown that CDK2-triggered histone gene transcription and unadulterated histone mediated histone mRNA degradation work together to regulate histone levels, thereby regulating DNA replication [46]. On the other hand, p21 inhibits CDK2, thereby arresting the cell cycle in the G1 phase [47]. Our studies demonstrated that DOR induced G1 phase cell cycle arrest in Mz-ChA-1 cells, preventing cell entry into the S phase by up-regulating p21 and down-regulating cyclin E1 and CDK2. These data suggest that cyclin E1 and CDK2 may be the main targets of DOR’s antiproliferative effects in Mz-ChA-1 cells.

Apoptosis inhibition or resistance is crucial in the process of carcinogenesis [48,49,50]. There are two main pathways of apoptosis: the intrinsic and extrinsic pathways [51,52]. Flow cytometry revealed a dose-dependent increase in apoptosis among Mz-ChA-1 cells treated with DOR. The mRNA and protein expression analysis showed that DOR mainly acts through the intrinsic apoptosis pathway, which may be related to DOR inhibiting BCL-2 expression [53]. DOR enhanced the intracellular BAX/BCL-2 ratio and activated the cleavage of caspase 3 and caspase 7. This conclusion was further confirmed by inhibitor pretreatment experiments.

ROS are pivotal in cellular functions and can initiate apoptosis via multiple pathways [54,55]. In this study, intracellular ROS levels showed a significant increase following DOR treatment. Previous studies have shown a robust link between intracellular ROS accumulation and cellular proliferation as well as apoptosis [54]. When ROS levels become excessive, they can lead to mitochondrial dysfunction [56]. Flow cytometry confirmed that DOR triggered a significant decrease in mitochondrial membrane potential (ΔΨm), resulting in mitochondrial dysfunction.

**Figure 6:**
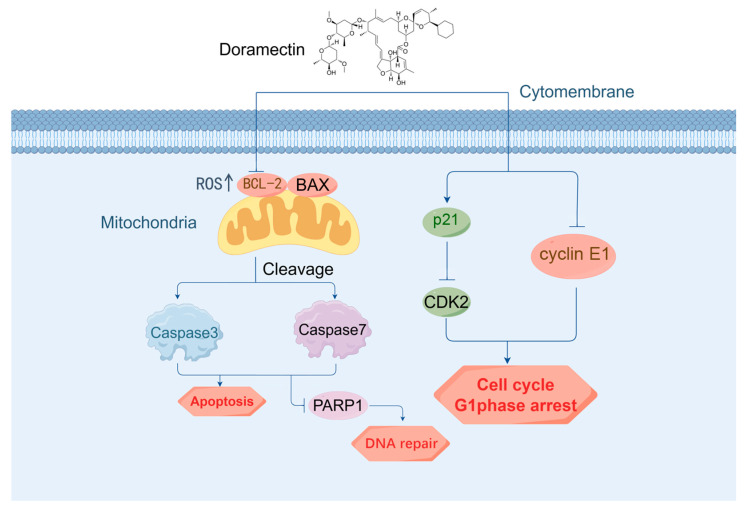
Overview of the mechanism of action of DOR.

In summary, DOR suppresses Mz-ChA-1 cell proliferation by causing G1 phase cell cycle arrest and enhancing apoptosis via the ROS-dependent mitochondrial pathway. (Figure 6). Future studies should focus on investigating the roles of differentially expressed genes to better understand the mechanisms underlying DOR’s biological effects, as well as its in vivo anticancer properties. These findings provide a strong foundation for further preclinical and clinical studies to assess DOR’s potential as a therapeutic option for this challenging cancer type.

## 4. Materials and Methods

### 4.1. Cell Culture

The human cholangiocarcinoma cells Mz-ChA-1 and QBC939 were donated by Professor Chundong Yu’s laboratory (Xiamen University, Xiamen, China). Routine mycoplasma testing of Mz-ChA-1 cells was performed using the Mycoplasma PCR Detection Kit (Beyotime Biotechnology, Shanghai, China) and they were found to be mycoplasma-negative. The cells were cultured in RPMI-1640 medium (Gibco, Life Technologies, Carlsbad, CA, USA) supplemented with 10% fetal bovine serum (ABW, Shanghai, China), 100 U/mL penicillin and 100 μg/mL streptomycin. Cells were cultured at 37 °C with 5% CO_2_. DOR (MCE, Monmouth Junction, NJ, USA) was dissolved in dimethyl sulfoxide (DMSO) and then diluted to various concentrations in the prepared cell culture medium for treatment.

### 4.2. Cell Viability Assay

Cell viability was assessed using the Cell Counting Kit-8 (CCK-8, Beyotime Biotechnology). Cells were plated at 5 × 10^3^ cells/well in a 96-well plate and treated with different concentrations of DOR (0, 5, 10, 15 μmol/L). After 24 or 48 h, 10 µL CCK-8 (diluted in 100 mL cell culture medium) was added to each well. The absorbance at 450 nm was measured using a POLARstar Omega automated multifunctional microplate reader (BMG LABTECH, Ortenberg, Germany).

Cell viability (%)=(Asample−Ablank)/(Acontrol−Ablank)×100%

### 4.3. LDH Release Test

Cells were incubated with varying doses of DOR for 48 h. LDH levels were determined with an LDH Cytotoxicity Assay Kit (Beyotime Biotechnology), according to the manufacturer’s instructions. After incubation, the absorbance at 490 nm was measured using a POLARstar Omega automatic multifunctional microplate reader (BMG LABTECH). Each experiment was performed in quadruplicate to ensure the consistency and reliability of the results.

LDH activity (relative fold change)=(ODsample/ODcontrol)×100%

### 4.4. Cell Colony Formation Assay

Cells were plated at 100 cells/well in 6-well plates. Control wells were incubated with fresh medium, whereas experimental wells were exposed to DOR at 5, 10 and 15 μmol/L. Following a 10-day incubation, cells were fixed in methanol for 15 min and stained with 0.1% crystal violet. Subsequently, the stained cells were counted to evaluate colony formation.

### 4.5. Transwell Cell Invasion Assay

After 48 h DOR treatment, cells were trypsinized and prepared into a cell suspension at the same concentration. The matrix gel was prepared following the Biozellen^®^ 3D Cell Culture Matrix Gel Kit protocol (Biozellen, Ord, NE, USA). After solidification, 0.1 mL of serum-free suspension (5 × 10^4^ cells/well) was placed in the Transwell’s upper chamber (Corning, NY, USA), with 0.8 mL complete medium in the lower chamber as a chemoattractant. Mz-ChA-1 cells that maintained their migratory and invasive capabilities could migrate and invade through the matrix gel by secreting matrix proteases, moving from the upper chamber to the lower chamber. The cells were incubated at 37 °C for 24 h. After incubation, the cells were fixed in 75% ethanol at room temperature for 15 min, then stained with 0.5% crystal violet for 30 min. The cells were dried, and the invasive cells were observed and recorded using an inverted phase contrast microscope.

### 4.6. Flow Cytometric Analysis of Apoptosis and Cell Cycle Distribution

Cell apoptosis was evaluated using the Annexin V-FITC and Propidine Iodide (Annexin V-FITC/PI) Apoptosis Detection Kit (Beyotime Biotechnology) and analyzed using flow cytometry. Cells were seeded in six-well plates and treated with DOR for 48 h. After incubation, cells were trypsinized and suspended in binding buffer. Annexin V-FITC (5 μL) and PI (5 μL) were added, followed by a 15 min incubation at room temperature in the dark. Apoptosis was assessed with a FC500 flow cytometer (Beckman Coulter, Brea, CA, USA). The data obtained were analyzed to quantify apoptotic cell percentages.

Mz-ChA-1 cells were exposed to DOR for 24 h. Fixed with 75% ethanol, the cells were resuspended and incubated with 5 μL of 10 mg/mL RNase at 37 °C for 30 min to degrade RNA. Subsequently, cells were stained with 5 μL of 10 mg/mL PI at 4 °C for 30 min in the dark to visualize DNA. The DNA content was analyzed using flow cytometry using an FC500 flow cytometer. The cell cycle distribution was analyzed using ModFit LT 5.0 software, and the percentage of cells in each phase (G0/G1, S and G2/M) was calculated to generate a cell cycle histogram.

### 4.7. Determination of ROS Production and Mitochondrial Membrane Potential

Cells were plated into 6-well plates and allowed to adhere for 12 h before being treated with varying concentrations of DOR for 48 h. After treatment, the cells were collected and incubated with 10 μM DCFH-DA (Beyotime Biotechnology) at 37 °C for 30 min in the dark. DCFH-DA is a fluorescent probe that measures ROS levels. Following incubation, the cells were collected and analyzed using an FC500 flow cytometer. The fluorescence intensity of DCF was used to assess ROS production in the cells.

Mitochondrial membrane potential (ΔΨm) assay: For mitochondrial membrane potential (ΔΨm) analysis, the treated cells were collected and incubated with JC-1 dye (Beyotime Biotechnology) for 1 h at 37 °C. JC-1 is a mitochondrial potential-sensitive dye that accumulates in healthy mitochondria, emitting red fluorescence, while it remains in the monomeric green form in depolarized mitochondria. After incubation, the cells were analyzed using an FC500 flow cytometer. The ratio of red/green fluorescence was used to assess the changes in ΔΨm, with a decreased red/green fluorescence ratio indicating mitochondrial depolarization.

### 4.8. DCFH-DA Fluorescent Probe Assay

Mz-ChA-1 cells were treated with different concentrations of DOR for 48 h. Cells were collected at the time point of arrival. The DCFH-DA probe from the kit was diluted with serum-free medium to prepare the working solution at a dilution ratio of 1:1000. The collected cells were then resuspended with the working solution, using approximately 300 µL of working solution per tube of cell sample. The samples were incubated uncapped in a 37 °C incubator for 20 min, preferably resuspended several times during the incubation period. At the end of the incubation, the cells were washed 3 times to remove excess DCFH-DA to prevent a very high background. Finally, cells were resuspended in 500 µL basal medium and assayed with a FC500 flow cytometer.

### 4.9. SDS–PAGE and Western Blot Analysis

Cells were plated in 12-well plates and incubated overnight to allow adhesion. After 24 h treatment with DOR or transfection with the appropriate constructs, the cells were lyzed in 2× loading buffer, and proteins were resolved by SDS–PAGE before being transferred to PVDF membranes (Millipore, Burlington, MA, USA). The membranes were blocked with 5% BSA for 1 h and incubated with primary antibodies (as listed in Table 1) overnight at 4 °C. After washing the membranes three times with 1× TBST, they were incubated with secondary antibodies (anti-rabbit IgG or anti-mouse IgG, ABclone, Wuhan, China) conjugated to HRP for 2 h at room temperature. Following further washes, protein bands were visualized using a gel imaging system (Azure Biosystems C280, Dublin, CA, USA), as per the manufacturer’s protocol. This method enables the detection and analysis of protein expression levels in response to treatments or transfections.

### 4.10. Real-Time Fluorescence Quantitative PCR (qPCR)

Total RNA was extracted using TRIzol reagent (Thermo Fisher Scientific, Waltham, MA, USA), according to the manufacturer’s instructions. The RNA was then reverse transcribed into complementary DNA (cDNA) using a reverse transcription kit. qRT-PCR was performed using the CFX384 Touch system (Bio-Rad, Hercules, CA, USA) and the Genious 2× SYBR Green kit (ABclone). The primers for qRT-PCR, which were validated for specificity and efficiency, were obtained from Sangon Biotech (Shanghai, China) and are listed in Table 2. GAPDH was used as the reference gene for normalization. The relative gene expression levels were calculated using the ΔCt method, allowing for the quantification of mRNA expression levels of the target genes in the treated samples compared to controls.

### 4.11. Transcriptome Analysis

Purified total RNA was extracted from Mz-ChA-1 cells treated for different durations using TRIzol reagent (Thermo Fisher Scientific). The RNA samples were then submitted to Majorbio (Shanghai, China) for transcriptome sequencing (RNA–Seq) analysis. The sequencing was conducted using the NovaSeq X Plus platform (Illumina, San Diego, CA, USA). For gene expression quantification, the fragments per kilobase of transcript per million fragments mapped (FPKM) method was employed. Differentially expressed genes (DEGs) were identified based on the following criteria: a log2 fold change > 1 and a Q-value < 0.001. The analysis of the DEGs included generating heatmaps of gene expression to visualize the patterns, performing GO enrichment analysis to identify biological processes, molecular functions, and cellular components associated with the DEGs, and conducting KEGG pathway analysis to explore the pathways enriched in the DEGs.

### 4.12. Statistical Analysis

Experiments were conducted in triplicate or quadruplicate. Data are expressed as mean ± SD. Statistical comparisons were made using the Student’s *t*-test for two-group analysis or a one-way ANOVA with Dunnett’s post hoc test for multiple comparisons. GraphPad Prism 9 (GraphPad Software, San Diego, CA, USA) was utilized for all statistical calculations. Significance levels were categorized as: ns (not significant) for *p* > 0.05, * for *p* < 0.05, ** for *p* < 0.01, *** for *p* < 0.001 and **** for *p* < 0.0001.

## Figures and Tables

**Table 1:** Types and sources of primary antibodies.

Name (Antibody)	Source
GAPDH	Proteintech, Rosemont, IL, USA
CDK2	ABclonal, Wuhan, China
Cyclin E1	ABclonal, Wuhan, China
p21	ABclonal, Wuhan, China
PARP1	ABclonal, Wuhan, China
BAX	ABclonal, Wuhan, China
BCL-2	Beyotime Biotechnology, Shanghai, China
Caspase 7	PTM BIO, Hangzhou, China
Caspase 3	ABclonal, Wuhan, China
Active caspase 3	ABclonal, Wuhan, China

**Table 2:** The primers used for qRT–PCR.

Name	Primer Sequences (5′-3′)
GAPDH	F: AGGTCGGAGTCAACGGATTT R: GCCATGGGTGGAATCATATTGG
CDK2	F: TTGGAGTCCCTGTTCGTACT R: GCGAGTCACCATCTCAGCAA
cyclin E1	F: GCAGCCAAACTTGAGGAAATCTA R: AATAGTCAGGGGACTTAAACGCCA
cyclin E2	F: GCTCCTAAAGTTCTTCTACCTCAGT R: GTAAAATGGCACAAGGCAGCA
p21	F: CTGTCTTGTACCCTTGTGCCTC R: TGGAGTGGTAGAAATCTGTCATGCT
BAX	F: CATGGGCTGGACATTGGACT R: GAGAGGAGGCCGTCCCAA
BCL-2	F: ATGTGTGTGGAGAGCGTCAA R: GGGCCGTACAGTTCCACAAA

## Data Availability

The datasets generated and analyzed during the current study are available in the NCBI SRA database under Bioproject # PRJNA1155816, Biosamples # SAMN43373319-SAMN43373324.

## Supplementary Materials

The following supporting information can be downloaded at: https://www.mdpi.com/article/10.3390/ijms252413440/s1.
