# Supplementary material for: Apoptosis-Inducing and Proliferation-Inhibiting Effects of Doramectin on Mz-ChA-1 Human Cholangiocarcinoma Cells
Source: Int J Mol Sci. 2024 Dec 15;25(24):13440. doi: 10.3390/ijms252413440 (PMC11676298; doi:10.3390/ijms252413440)
Supplement: Supplementary file 1 [file ijms-25-13440-s001.zip › ijms-3351996-supplementary.pdf]

Supplementary Figures

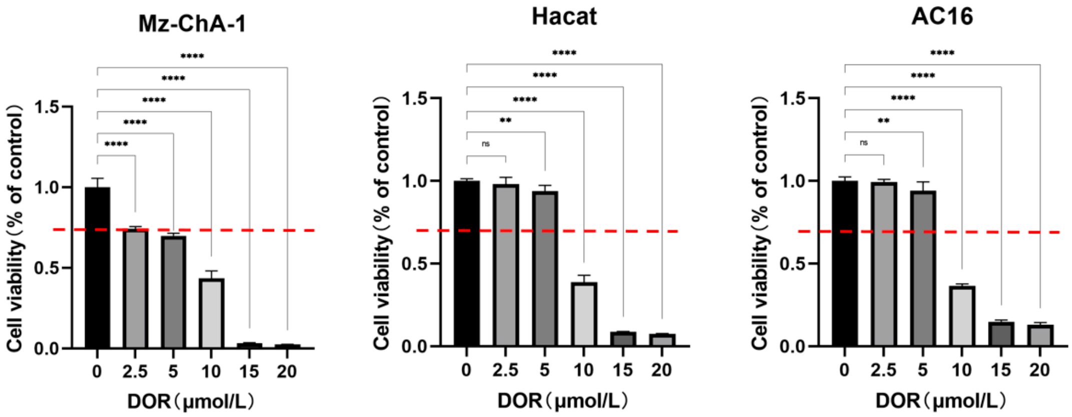

Figure S1. Inhibition efficiency of DOR in normal cell lines. Mz-ChA-1, Hacat and AC16 cells were treated with different concentrations of DOR for 48 h, respectively, and the bar graphs show the cell viability of different cell lines at the corresponding concentrations ($n = 5$). The red dotted line represents a cell survival rate of 74.18%. Significance levels were categorized as: ns: not significant, $p > 0.05$, ** $p < 0.01$, **** $p < 0.0001$.
